# PHYSICAL ACTIVITY AND EPISODIC MEMORY IN AFRICAN AMERICAN OLDER ADULTS

**DOI:** 10.1093/geroni/igac059.1904

**Published:** 2022-12-20

**Authors:** Rio Tate, Brent Small

**Affiliations:** University of South Florida, Tampa, Florida, United States; University of South Florida, Tampa, Florida, United States

## Abstract

African Americans are more likely to have Alzheimer’s Disease (AD) yet less likely to be included in AD research. Additionally, memory complaints may signal the clinical genesis of AD. Interventions such as physical activity that can help individuals maintain their cognitive functioning as they age have merit in research. The objective of this study is to examine the influence of physical activity of various intensities on episodic memory in African American older adults at the interindividual and intraindividual levels over time. We used data from the Health and Retirement Study over 12 years. Our analyses included an indicator of episodic memory and we examined physical activity of three intensities as predictors while controlling for relevant demographic and health variables. Preliminary data indicates that African American older adults who engaged in more frequent physical activity (regardless of intensity) had better episodic memory. Individuals who engaged more frequently in physical activity had a slightly greater rate of decline than those who engaged less frequently in physical activity. Physical activity may act as a buffer against cognitive decline for older adults. Intensity need not be vigorous to observe changes.

